# Specialized Infrared Camera-Computer System (SPY) Imaging for Monitoring the Restoration of Nipple Sensation in Patients Undergoing Breast Reduction

**DOI:** 10.7759/cureus.72880

**Published:** 2024-11-02

**Authors:** Brian Celso, Nicole Murray, John Murray

**Affiliations:** 1 Surgery, University of Florida College of Medicine – Jacksonville, Jacksonville, USA; 2 Surgery, Huntsman Cancer Institute, University of Utah, Salt Lake City, USA; 3 Plastic Surgery, University of Florida College of Medicine – Jacksonville, Jacksonville, USA

**Keywords:** breast reduction surgery (brs), nipple areolar complex, nipple sensation, spy, symptomatic mammary hypertrophy

## Abstract

Introduction

Symptomatic mammary hypertrophy (SMH) refers to excessive breast weight exceeding 3% of total body weight, impacting not only the breast but also the nipples and areola. Breast reduction surgery (BRS) has a complication that adversely affects the nipple-areolar complex (NAC) sensation. The purpose of this study was to estimate the degree to which the specialized infrared camera-computer system (SPY) may predict postoperative sensation of the NAC following BRS.

Methods

A retrospective, observational study that included 408 SMH patients who underwent BRS was conducted at the Division of Plastics Surgery at the University of Florida College of Medicine - Jacksonville. Breast surgery patients were grouped according to whether SPY was used intraoperatively (SPY group) or not used intraoperatively (NOSPY group) during surgery. A chi-square test was used to evaluate whether a percentage difference existed between the SPY group and the NOSPY group. The main outcomes were unchanged, decreased, or increased NAC sensation. An Eta square was used to measure the effect sizes of profusion variance associated with reported nipple sensation. An area under the curve (AUC) was performed to determine the most favorable cutoff for SPY profusion sensitivity and specificity. A nominal regression model was used to determine the correlation between NAC sensation and SPY usage. A probability value less than 0.05 was considered statistically significant.

Results

Of the 408 SMH patients included in the study, SPY technology was incorporated for 63 patients (15.4%). The percentage of those who reported decreased nipple sensation with the use of SPY was 29 (47.6%). The percentage of those who reported increased nipple sensation with the use of SPY was 4 (6.4%). The percentage of patients who reported no change in nipple sensitivity with the use of SPY was 29 (46.0%). The chi-square test was statistically significant (χ2 = 302.29, df = 2, p < 0.001). The Eta squared for the right breast SPY profusion percent was 0.74 and for the left breast SPY profusion percent was 0.81. Both percentages represent large effect sizes as the proportion of variance associated with the reported nipple sensation. The AUC for the SPY profusion was 0.471, which was not statistically significant. The most favorable receiver operator characteristic curve SPY profusion sensitivity was 0.651, with a specificity of 0.690 and an associated cutoff of 65.5. The outcome variable, NAC sensation, was determined to be significantly correlated with SPY use predicting decreased and increased NAC sensitivities (OR 3.6; p < 0.001 and OR 6.4; p = 0.007), respectively.

Conclusions

Although SPY technology has traditionally been utilized to assess tissue perfusion, our study demonstrates its potential as a predictive tool for postoperative NAC sensation.

## Introduction

Symptomatic mammary hypertrophy (SMH), also known as macromastia, is the excess breast weight that exceeds 3% of the total body weight [1]. The condition is characterized by an oversized breast size, which can cause physical discomfort, pain, and emotional distress. Possible causes of SMH include genetics, hormonal imbalances, such as excessive estrogen or growth hormone levels, and puberty, when breast tissue grows rapidly. Significant weight gain and certain medical conditions, such as thyroid disorders, can also contribute to breast hypertrophy. Hypertrophy of the breast can affect the nipples and areola in addition to the breast tissue.

The primary nerve supply of the nipple-areolar complex (NAC) is the medial, lateral, and central branches, which run alongside vessels supplying the nipple [2]. The primary blood supply of the NAC is provided by branches of the internal mammary and lateral thoracic arteries. The remaining blood flow to the NAC relies on the subdermal plexus, which forms an anastomotic network encircling the areola from both medial and lateral sources [3]. A peri-areolar incision interrupts this network, diminishing the blood supply. As nerves and vessels often travel together, higher tissue perfusion - as detected by SPY technology - may be linked to better nerve preservation [4]. Indices such as monofilament testing, pressor sensory devices, and QOL surveys are valuable tools to establish benchmarks for optimal patient satisfaction and as a means of assessing surgeon performance.

Breast reduction surgery (BRS) is a common treatment option for mammary hypertrophy. There are, however, risks and complications associated with BRS. Among these is nipple sensation that may be reduced or lost due to nerve damage. Unfortunately, BRS has been known to adversely affect sensation (NAC) [5]. Recent literature depicts how surgical techniques may be associated with improved return of nipple and skin sensation post-mastectomy [6]. A two-fold return of nipple sensation was found for peri-areolar incision vs. upper outer quadrant incision. Increased “time from surgery” is another factor reported in studies, which may be associated with improved return of sensation to the NAC [6-8]. To date, no studies have reported tools that predict nipple sensation.

A specialized infrared camera-computer system (SPY) is the current tool used to monitor intraoperative skin perfusion to the NAC prior to the termination of breast surgery [6-12]. The SPY fluorescence imaging system utilizes indocyanine green (ICG), a water-soluble dye that is injected intravenously, binds to plasma proteins, and fluoresces the vascular system [13]. Due to its short half-life and low toxicity, ICG may be used for sequential imaging [6]. When assessing blood flow to the NAC, relative perfusion is determined by the level of ICG detected within the tissue [14]. Specifically, gray SPY imaging represents a low level of ICG, while red SPY imaging represents a high level of ICG and thus better blood flow at the NAC. The aim of our study was to analyze the use of SPY during BRS to predict postoperative nipple sensation reported as decreased, increased, or unchanged.

## Materials and methods

The research setting was the University of Florida College of Medicine - Jacksonville campus, which is located in the city’s urban core and serves as the region’s safety net hospital. The University of Florida Institutional Review Board acted as the approval body for this study (IRB201800521). This was a retrospective observational research design of electronic health records. Patient demographics collected included age, ethnicity, BMI, and tobacco use. The resection weight in grams of the left and right breasts was also recorded. Breast surgery patients were then grouped according to whether SPY was used intraoperatively (SPY group) or not used intraoperatively (NOSPY group) during surgery. The primary outcome of the analysis was the reported nipple sensation following BRS (decreased, increased, or unchanged).

Study population

The study population included women 18 years and older who were treated for SMH by the plastic surgery department. Exclusion criteria were non-English speaking patients.

Procedure

Spy angiography was performed in all cases to assess perfusion to the NAC (except in patients who were allergic to iodine) during BRS. If the perfusion was less than 20%, the NAC would have been grafted. SPY findings did not change the procedure in such cases (i.e., no nipples were grafted due to the low perfusion index). Nipple sensation change was based on the self-report of either decreased, increased, or normal. Evaluation of nipple sensation occurred within the six-month postoperative period after BRS.

Statistical analysis

Descriptive summaries in the form of frequencies and percentages were reported for categorical variables, while means and standard deviations were reported for numeric variables. Parametric and nonparametric tests were also used as appropriate. A chi-square test was used to evaluate differences in the proportions of nipple sensation change between whether SPY was used intraoperatively or not used intraoperatively. A t-test was used to evaluate whether a difference in nipple sensitivity existed between the SPY group and the NOSPY group. Eta square was used to measure the effect sizes of profusion variance associated with reported nipple sensation. An area under the curve (AUC) was performed to determine the most favorable cutoff for SPY profusion sensitivity and specificity.

A nominal regression model was then utilized to measure the strength and direction of the associations that exist between the predictor variables age, BMI left and right breast perfusion, tobacco use history, and SPY usage with the categorical outcome variable, nipple sensation, determined as decreased, increased, or normal. The normal sensation group was used as the comparator in the model. Both SPY and NOSPY groups were included in the same model to reduce the risk of an increased family-wise error rate when using multivariate outcomes. The differences were estimated using least-square means, which are model-based predictions of the marginal means, often called “adjusted means.” Covariates that were adjusted for the nominal regression model were age, BMI, tobacco use, and resection weight in grams. The level of significance was set at 0.05. All analyses were performed in IBM SPSS Statistics for Windows, Version 29.0 (Released 2022; IBM Corp., Armonk, NY, USA) [15].

## Results

A total of 408 medical records were included for review in this study. The demographic statistics of the participants are presented in Table 1. The average resection weight of the right breast was 827.57 grams (SD = 459.09) and of the left breast was 818.02 grams.

**Table 1:** Demographic statistics

Variable	n	Mean	SD
Age	422	37.7	12.9
BMI	422	35.8	6.8
Race, n (%)			
Black	328 (75.4%)	-	-
White	83 (19.1%)	-	-
Hispanic	21 (4.8%)	-	-
Asian	2 (0.5%)	-	-
Middle Eastern	1 (0.2%)	-	-
Tobacco use, n (%)			
Never	301 (71.3%)	-	-
Active	9 (2.1%)	-	-
Former	112 (26.6%)	-	-

The number of breast reduction surgeries that incorporated SPY technology was 63 (15.4%) and NOSPY was 359 (84.6%). Of the total patients in the study, 106 (26.0%) reported decreased nipple sensation. The number of patients who reported increased nipple sensation was 10 (2.5%), while those who reported unchanged nipple sensation were 292 (71.6%). The chi-square test was statistically significant (χ2 = 302.29, df = 2, p < 0.001). There was no statistical difference in nipple sensitivity between the SPY group versus the NOSPY group (t = 0.773, p = 0.441).

**Figure 1:**
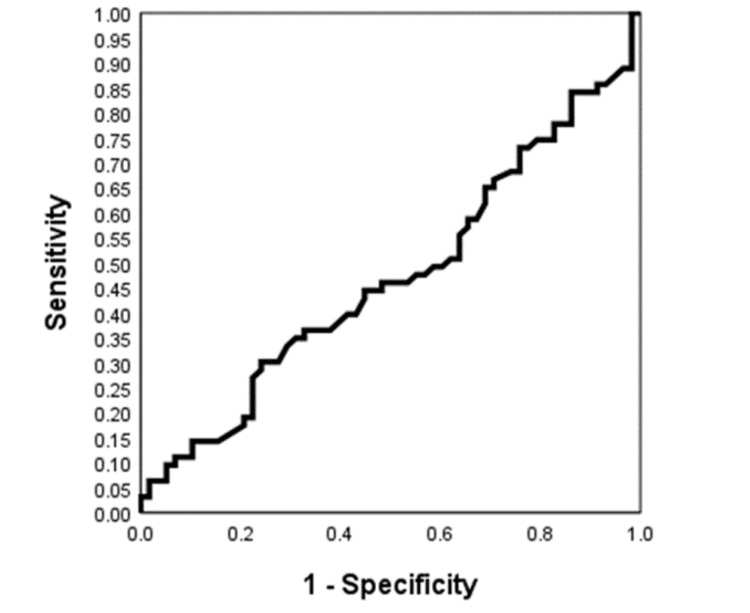
SPY ROC curve Diagonal segments are produced by ties. ROC, receiver operator characteristic

For the SPY group, the percentage of patients who reported decreased nipple sensation was 29 (47.6%). The percentage of those who reported increased nipple sensation with the use of SPY was 4 (6.4%), and the percentage of patients in the SPY group who reported no change in nipple sensitivity was 29 (46.0%). The chi-square test was statistically significant (χ2 = 20.16, df = 2, p < 0.001). The Eta squared for the right breast SPY profusion percent was 0.74 and for the left breast SPY profusion percent was 0.81. Both percentages represent large effect sizes as a measure of the proportion of variance associated with the reported nipple sensation. The AUC for the SPY profusion was 0.471, which was not statistically significant. The most favorable receiver operator characteristic curve SPY profusion sensitivity was 0.651, with a specificity of 0.690 and an associated cutoff criterion of 65.5 shown in Figure 1.

The nominal regression model factored whether or not SPY was used during surgery and NAC sensitivity as the outcome variable. The covariates included in the model were age, BMI, tobacco use, and resection weight to control for their individual effects. For the purposes of this study, unchanged NAC sensitivity and NOSPY served as the reference groups. The final model fit index was statistically significant (χ2 = 46.02, df = 12, p < 0.001). The parameter estimates are displayed in Table 2.

**Table 2:** Nominal regression model ^a^ The reference category is “Never used tobacco.” ^b^ The reference category is “Normal nipple sensation.” ^*^ Significance at 0.01 ^**^ Significance at 0.001

Parameter estimates	Beta	OR (95% CI)	p-value
Decreased nipple sensation			
Age	-0.035	0.966 (0.946-0.986)	0.001^**^
BMI	0.026	1.026 (0.983-1.072)	0.234
Right side breast	0	1.000 (0.999-1.002)	0.817
Left side breast	0	1.000 (0.999-1.002)	0.883
Tobacco use^a^	-0.273	0.761 (0.461-1.257)	0.286
SPY use^b^	1.292	3.641 (2.013-6.584)	<0.001^**^
Increased nipple sensation			
Age	-0.003	0.997 (0.946-1.050)	0.905
BMI	-0.057	0.945 (0.834-1.070)	0.372
Right side breast	-0.002	0.998 (0.993-1.003)	0.489
Left side breast	0.002	1.002 (0.997-1.006)	0.538
Tobacco use^a^	-0.782	0.457 (0.092-2.280)	0.34
SPY use^b^	1.854	6.384 (1.670-24.413)	0.007^*^

The outcome variable, NAC sensation, was determined to be associated with SPY use vs. NOSPY use with decreased and increased NAC sensitivities (p < 0.001 and p = 0.007), respectively, compared to the unchanged NAC sensation group. Age was found to be statistically significant for reported decreased NAC sensation while controlling for all other variables (Table 2). The OR for nipple sensitivity change among SPY patients was a 3.6-fold reported decreased NAC sensitivity and 6.4 times reported increased NAC sensitivity for those SPY patients when compared to the NOSPY group and to patients with unchanged NAC sensation even after controlling for all other variables.

## Discussion

Nipple sensitivity is an important consideration for patients undergoing BRS due to SMH. Awareness and understanding of the potential changes in nipple sensation, coupled with effective management and prevention strategies, can significantly enhance patient outcomes and satisfaction. In our retrospective cohort study, the use of SPY technology during surgery was significantly correlated with detecting a change in NAC sensation. Our study also revealed that a model based on patient demographics and profusion was able to predict the odds of a decrease in nipple sensitivity. Finally, a SPY cutoff score was computed that offered the best likelihood of change in nipple sensation. Unfortunately, the resultant cutoff score was no better than chance.

Few studies have remarked on peri-surgical techniques that have improved postoperative NAC sensation. A meta-analysis that analyzed studies that assessed nipple sensitivity preservation due to ancillary perioperative technique showed promising results. For example, Djohan et al. [16] reported that 100% of patients (n = 3 breasts) with concurrent peri-operative neurotization had better sensation in six of eight NAC areas postoperatively, compared to breasts that had not undergone neurotization. Even fewer studies have proposed quantitative measures to assess NAC sensation postoperatively. Most studies published have reported utilizing a combination of monofilament testing and questionnaires [7,17,18]. Other studies have assessed NAC sensory thresholds using the pressure-specified sensory device [19,20]. Our study is unique in that it proposes an innovative method to predict nipple sensation perioperatively, which may aid surgeons in optimizing techniques to achieve better postoperative nipple sensation for patients.

A study of 274 breast reduction patients revealed that those who received a vertical pattern incision had decreased NAC sensation compared to those who underwent a wise pattern incision (26% vs. 13%; p = 0.0025) [21]. Furthermore, patients who experienced minor complications such as infections resolved with oral antibiotics or successfully treated with sharp debridement also reported decreased NAC sensation following surgery compared to those without minor postoperative complications (23% vs. 15%; p = 0.0264) [21]. Past research on breast cancer patients undergoing total skin-sparing mastectomy found that incisions extending more than 30% of the circumference of the areolar were independently and statistically associated with a higher risk of nipple necrosis (p < 0.001) [22]. Moreover, patients undergoing nipple-sparing mastectomy (NSM) showed that peri-areolar incisions significantly increased the odds patients were susceptible to nipple ischemia (OR 9.69, p = 0.014) [23].

Breast reconstructive surgery studies have attributed peri-surgical factors such as the degree of reconstructive surgery or type of reconstructive tissue used to a decreased NAC sensation following breast reconstruction [8,24]. One retrospective cohort study of 349 patients who had undergone NSM reported that following implant reconstruction, 48% of patients had decreased breast sensation, and 57% had decreased nipple sensation [8]. Similarly, a retrospective cohort study of 127 breast cancer patients undergoing mastectomy with immediate breast reconstruction found that patients undergoing reconstruction with abdominal tissue as opposed to prosthetic reconstruction were more likely to experience significantly decreased NAC sensation (OR, 0.270; p = 0.004) [24].

Strengths and limitations

To our knowledge, this is the first study to assess the intraoperative use of SPY technology to predict post-breast reduction NAC sensation. Our results imply that the adoption of SPY technology during BRS may assist surgeons in refining techniques to improve nipple sensation in patients postoperatively. Also, since our study was performed by a single experienced surgeon, there was no variability in measured NAC sensation, thus reducing information bias. This also eliminated the confounding factors of training, experience, technique, and expertise differences among providers. However, there were several limitations in the present study that made the conclusions tentative. The first limitation was the retrospective nature of the study design, which exposes the results to biases inherent in such cohort studies. Additionally, a standardized sensation scale was not utilized in the evaluation of BRS patients postoperatively, which predisposed the study to recall bias with regard to self-reported NAC sensation. The second limitation was the limited dataset that was collected at a single urban center, a safety net hospital. Thus, the ability to generalize the results may be restricted to similar patients.

## Conclusions

The use of SPY as a tool for perfusion analysis may increase the odds of enhancing the preservation of intact nipple-areolar sensation. However, follow-up studies are needed to evaluate more long-term benefits. While the surgery significantly improves the quality of life for many patients, it can have complex postoperative outcomes, one of which is nipple sensitivity. Collaborating with an experienced surgical team and adhering to postoperative care instructions are paramount in achieving optimal results and ensuring a smooth recovery process. 
